# Predictors of HIV Testing among Youth in Sub-Saharan Africa: A Cross-Sectional Study

**DOI:** 10.1371/journal.pone.0164052

**Published:** 2016-10-05

**Authors:** Ibitola O. Asaolu, Jayleen K. Gunn, Katherine E. Center, Mary P. Koss, Juliet I. Iwelunmor, John E. Ehiri

**Affiliations:** 1 Department of Health Promotion Sciences, Mel and Enid Zuckerman College of Public Health, University of Arizona, 1295 N Martin Avenue, Tucson, Arizona, 85724, United States of America; 2 Department of Epidemiology and Biostatistics, Mel and Enid Zuckerman College of Public Health, University of Arizona, 1295 N Martin Avenue, Tucson, Arizona, 85724, United States of America; 3 Department of Obstetrics and Gynecology, University of Arizona, 1501 N Campbell Ave, Tucson, Arizona, 85724, United States of America; 4 Department of Kinesiology and Community Health, University of Illinois at Urbana Champaign, 1206 S. 4th Street, Champaign, Illinois, 61820, United States of America; UCL Institute of Child Health, University College London, UNITED KINGDOM

## Abstract

**Introduction:**

In spite of a high prevalence of HIV infection among adolescents and young adults in sub-Saharan Africa, uptake of HIV testing and counseling among youth in the region remains sub-optimal. The objective of this study was to assess factors that influence uptake of HIV testing and counseling among youth aged 15–24 years in sub-Saharan Africa.

**Methods:**

This study used the Demographic and Health Survey (DHS) data from countries that represent four geographic regions of sub-Saharan Africa: Congo (Brazzaville), representing central Africa (DHS 2011–2012); Mozambique, representing southern Africa (DHS 2011); Nigeria, representing western Africa (DHS 2013); and Uganda, representing eastern Africa (DHS 2011). Analyses were restricted to 23,367 male and female respondents aged 15–24 years with complete data on the variables of interest. Chi-square tests and logistic regression models were used to assess predictors of HIV testing. Statistical significance was set at p< 0.01.

**Results:**

The analysis revealed that a majority of the respondents were female (78.1%) and aged 20-24-years (60.7%). Only a limited proportion of respondents (36.5%) had ever tested for HIV and even fewer (25.7%) demonstrated comprehensive knowledge of HIV/AIDS. There was a significant association between HIV testing and respondents’ gender, age, age at sexual debut, and comprehensive knowledge of HIV in the pooled sample. Older youth (adjusted OR (aOR) = 2.19; 99% CI = 1.99–2.40) and those with comprehensive knowledge of HIV (aOR = 1.98; 1.76–2.22) had significantly higher odds of ever being tested for HIV than younger respondents and those with limited HIV/AIDS knowledge respectively. Furthermore, men had lower odds of HIV testing than women (aOR = 0.32; 0.28–0.37).

**Conclusions:**

Reaching youth in sub-Saharan Africa for HIV testing continues to be a challenge. Public health programs that seek to increase HIV counseling and testing among youth should pay particular attention to efforts that target high-risk subpopulations of youth. The results further suggest that these initiatives would be strengthened by including strategies to increase HIV comprehensive knowledge.

## Introduction

Globally, significant progress has been made in efforts to address the burden of the Human Immuno-Deficiency Virus (HIV) and Acquired Immune-Deficiency Syndrome (AIDS), leading to a 38% reduction in new infections since 2001 [1]. In spite of this remarkable progress, HIV/AIDS remains a major global health problem [1]. According to the United Nations Joint Programme on HIV/AIDS (UNAIDS), 2.1 million people were newly infected with HIV in 2013, and an estimated 35 million people were living with the virus [2]. Specifically, there has been a decreasing trend in incidence of HIV infection among adolescents in many countries. After over three decades since the first case of HIV was discovered, there is evidence that overall, knowledge about the virus infection is increasing, leading to a trend towards less risky sexual health behaviors, and thus reduction in new infections [3]. In spite of this, HIV remains particularly devastating for adolescents and young adults aged 15–24 years who account for approximately 50% of all new HIV infections and 33% of persons living with HIV/AIDS worldwide [4]. Globally, there is an estimated 1.2 billion adolescents (10–19 year-olds), constituting 18% of the world’s population [5]. Available evidence shows that about 2.2 million of these (60% of them, female) are living with HIV [5], and many are unaware of their infection [6]. Adolescence and emerging adulthood constitute a period of transition, growth, exploration, and opportunities. During this phase of life, young people develop an increased interest in sex, with concomitant risks for sexually transmitted infections (STIs), including HIV. Furthermore, adolescence and emerging adulthood is known as a stage of development that is characterized by a high sense of invulnerability [7], which is often associated with increased risk taking [8, 9]. A study by Anderson and colleagues found an inverse relationship between perceived risk of HIV and sexual debut; youth with high levels of perceived risk of HIV had lower odds of having sexual intercourse compared to those with low perceived risk of HIV [9].

Sub-Saharan Africa is the region where persons aged 10–19 years constitute the largest proportion of the population [5]. Youth in this region are particularly at a heightened risk for HIV infection with 2.2% of young women and 1.1% of young men living with HIV as of 2013 [1]. According to available evidence, only 10% of young men and 15% of young women aged 15–24 years in sub-Saharan Africa knew their HIV status in 2013 [1]. Barriers to HIV testing include lack of awareness of available services, low perception of personal risk, fear of negative consequences associated with a positive test result (including stigma), concerns about confidentiality, financial burden of testing, and lack of HIV/AIDS knowledge [10–12]. These barriers have contributed to generations of individuals whose lives, dreams, and potential contribution to economic development have been undermined by a largely preventable disease. Young women in sub-Saharan Africa are particularly vulnerable to HIV infection; of all young women aged 15–24 living with HIV globally, 80% of them reside in sub-Saharan Africa [1, 9–12]. In many parts of sub-Saharan Africa, the HIV infection risk for adolescent girls is 2–6 folds higher than that of their male counterparts by the time they are aged 20–24 years [1]. The higher prevalence of HIV infection among young women is associated with lower levels of accurate HIV knowledge, age-disparate sexual relationships, gender-based violence, poverty, certain cultural practices, and early sexual debut [10–14].

Although it is recognized that this population has different needs from those of children and adults, there are few global initiatives that are specifically devoted to the promotion of HIV counseling, testing, and linkage to care among adolescents and young adults. The problem of limited initiatives is of major global concern because HIV counseling and testing is an important entry point for most HIV/AIDS care and prevention interventions [14]. Given the wider availability of antiretroviral therapy, efforts to increase uptake of HIV counseling and testing among youth have the potential to lead to early diagnosis, prompt linkage to care, improved treatment outcomes, and reduction in new infections [4].

It is important to understand and address the challenges that young people in sub-Saharan Africa face in accessing HIV counseling and testing, with the hope that such an understanding might help inform future research, practice, and policy regarding interventions that address young people’s unique barriers to HIV counseling and testing. The objective of this study was to assess factors that influence uptake of HIV testing among youth aged 15–24 years in sub-Saharan Africa.

## Methods

### Survey

This study used the Demographic and Health Survey (DHS) data from four countries that represent unique regions of sub-Saharan Africa: Congo (Brazzaville, DHS 2011–2012), representing Central Africa; Mozambique (DHS 2011), representing Southern Africa; Nigeria (DHS 2013), representing Western Africa; and Uganda (DHS 2011), representing Eastern Africa. The DHS is a nationally representative survey that uses a multistage and stratified design to collect information on population health, HIV/AIDS, malaria, and nutrition within each country [15]. The individual women’s and men’s data of the DHS dataset were used in this analysis. Only youth, defined by the United Nations “as individuals between the ages of 15 and 24 years” [16] were included in the analysis.

### Measures

The variable assessing comprehensive knowledge of HIV was created using UNICEF’s definition of comprehensive HIV knowledge [17]. Participants were classified as having comprehensive HIV knowledge if they correctly: 1) identified two methods of preventing sexual transmission of HIV; 2) acknowledged that a healthy looking person can have HIV; and 3) rejected two common misconceptions about HIV transmission, i.e., HIV can be transmitted through mosquito bites or by sharing food with an HIV infected person. The variable on having a history of sexually transmitted infection (STI) was classified as ‘yes’ if a respondent answered ‘yes’ to any of the following items: had any STI in last 12 months; had genital sore/ulcer in last 12 months; or had abnormal or bad smelling genital discharge in last 12 months [18]. History of STI was categorized as ‘no’ if the respondent answered ‘no’ to these questions. Urban versus rural residence (considered as a system-level predictor of HIV testing) was used as a proxy for access to health care facilities.

### Statistical Analysis

Due to oversampling of certain populations, country-specific individual weights were used in all analyses. Using weights makes the data more representative of the study population on a national level [15]. Chi-square tests and logistic regression models were used to assess predictors of HIV/AIDS testing. Statistical significance was reduced from p < 0.05 to p < 0.01 to control for type-1 error emanating from the large sample size of the DHS data. All data analyses were conducted on SAS 9.4 (Cary, North Carolina) using the ‘PROC SURVEY’ command. The Demography and Health Surveys were approved by the Institutional Review Board of ORC Macro. In addition, this secondary data-analysis was reviewed and approved as exempt by the Institutional Review Board of the University of Arizona. Participants' information were anonymized and de-identified prior to analysis.

## Results

### Pooled Sample

The analytic sample consisted of 23, 367 respondents including the following: 4,482 (19.2%) Congolese; 5,301 (22.7%) Mozambicans; 10,942 (46.8%) Nigerians; and 2,642 (11.3%) Ugandans. The demographic characteristics of respondents are presented in Table 1. The total sample consisted predominantly of females (78.2%) and youth aged 20 to 24 (60.7%). About half (50.2%) of the respondents had at least a secondary school education, and slightly more than half (58.1%) lived in rural areas. About 3 out of 5 respondents (62%) had no sexual partner other than their current partner or spouse, within the past year and did not report symptoms suggestive of an STI (88.1%) in the 12 months preceding the survey. Few (25.7%) demonstrated comprehensive knowledge of HIV/AIDS, and only a limited proportion (36.5%) had ever been tested for HIV.

**Table 1:** Demographic and health characteristics of participants.

	CONGO		MOZAMBIQUE		NIGERIA		UGANDA		TOTAL	
	n^a^	%^b^	n^a^	%^b^	n^a^	%^b^	n^a^	%^b^	n^a^	%^b^
**Sex of Respondents**										
Male	1228	28.3	1184	22.2	2256	19.6	521	18.7	5189	21.8
Female	3254	71.7	4117	77.8	8686	80.4	2121	81.3	18178	78.2
**Age of Respondents**										
15 to 19 years	2148	43.1	2449	46.1	3788	34.6	1000	38.0	9385	39.3
20–24 years	2334	58.9	2852	53.9	7154	65.4	1642	62.0	13982	60.7
**Age at Sexual Debut**										
Less than 15 years	1502	27.5	1492	28.4	2433	23.6	544	21.6	5971	22.3
15–18 years	2821	67.3	3509	65.5	6274	56.5	1724	65.1	14328	61.6
19 years and above	159	5.2	300	6.1	2235	19.9	374	13.3	3068	13.1
**Area of Residence**										
Urban	1642	71.5	2438	38.4	3731	34.9	950	25.2	8761	41.9
Rural	2840	28.5	2863	61.6	7211	65.1	1692	74.8	14606	58.1
**Education**										
None	226	3.2	689	15.7	3527	36.0	151	3.8	4593	21.4
Primary	1262	20.7	2617	52.3	1439	12.9	1420	57.1	6738	28.4
Secondary or higher	2994	76.1	1995	31.0	5976	51.1	1071	39.1	12036	50.2
**Wealth Index**										
Poorest	1483	14.0	649	17.8	1893	19.4	443	15.3	4468	17.5
Poorer	1240	20.3	750	17.7	2347	22.0	424	17.6	4761	20.2
Middle	696	22.4	890	17.8	2429	20.7	431	19.1	4446	20.2
Richer	592	22.6	1150	20.7	2403	20.1	483	19.4	4628	20.7
Richest	471	20.7	1862	26.0	1870	17.8	861	28.6	5064	21.4
**Comprehensive Knowledge of HIV**^c^										
No	3466	73.7	3799	74.4	8398	76.5	1781	66.4	17444	74.3
Yes	1016	26.3	1502	25.6	2544	23.5	861	23.5	5923	25.7
**Number of Sexual Partners (excluding spouse) in the past 12 months**										
None	1893	40.9	2971	60.2	7226	69.8	1825	70.6	13915	62.0
1 or more	2589	59.1	2330	39.8	3716	30.2	817	29.4	9452	38.0
**Sexually Transmitted Infection in Past 12 months**										
No	3745	81.0	4830	91.5	10140	92.6	2024	74.8	20739	88.1
Yes	737	19.0	471	8.5	802	7.4	618	25.2	2628	11.9
**Ever-Tested for HIV**										
No	3228	68.6	2631	54.7	8184	75.3	600	22.5	14643	63.5
Yes	1254	31.4	2670	45.3	2758	24.7	2042	77.5	8724	36.5
**Received Results of HIV Test**^d^										
No	120	6.6	201	8.4	370	13.7	107	5.1	798	9.0
Yes	1134	93.4	2469	91.6	2388	86.2	1935	94.9	7926	91.0
**TOTAL (N)**	4482		5301		10942		2642		23367	

Notes:

^“a”^ = Unweighted frequency of respondents

^“b”^ = Weighted percent

^“c”^ = Comprehensive knowledge assesses respondents’ understanding of HIV transmission and prevention.

^“d”^ = Adds up to the total number of people who tested for HIV

Table 2 presents results of the relationship between individual and system-level factors and uptake of HIV testing and counseling among youth in the study countries. HIV testing was significantly associated with respondents’ gender (p< 0.0001), age (p< 0.0001), age at sexual debut (p<0.0001), comprehensive knowledge of HIV (p<0.0001), number of sexual partners in the past year (p<0.0001), and history of STIs (p<0.0001). Male youth had a lower proportion (23.1%) of HIV testing than female youth (40.3%). Similarly, fewer youth (26.5%) aged 15 to 19 years tested for HIV than those aged 20–24 years (40.3%). More youth with comprehensive knowledge of HIV (50.3%) tested for HIV compared to those without comprehensive knowledge of the virus (31.8%). Among youth who reported a history of STI, 49.3% had tested for HIV compared to 34.8% of youth with no history of STI.

**Table 2:** Weighted Chi-square test of association between uptake of HIV testing and respondents’ characteristics.

	CONGO	MOZAMBIQUE	NIGERIA	UGANDA	TOTAL
	Yes (%)	Yes (%)	Yes (%)*	Yes (%)*	Yes (%)
**Sex of Respondents**					
Male	18.8	21.4	20.2	52.0	23.1
Female	36.4 ***	52.1 ***	25.8 ***	83.4 ***	40.3 ***
**Age of Respondents**					
15 to 19 years	22.4	33.2	13.9	66.0	26.5
20–24 years	38.3 ***	55.7 ***	30.4 ***	84.6 ***	43.1 ***
**Age at Sexual Debut**					
Less than 15 years	26.6	43.7	13.3	68.4	29.1
15–18 years	32.3	45.3	24.9	79.2	37.8
19 years and above	45.6 **	52.8	37.8 ***	84.3 ***	45.2 ***
**Area of Residence**					
Urban	34.7	54.7	38.8	82.7	43.6
Rural	23.2 ***	39.4 ***	17.2 ***	75.8 **	31.4 ***
**Education**					
None	15.6	35.8	6.2	69.7	12.7
Primary	21.5	41.0	22.9	73.3	41.5
Secondary or higher	34.8 ***	57.5 ***	38.2 ***	84.5 ***	43.9 ***
**Wealth Index**					
Poorest	17.1	28.9	4.8	79.8	19.5
Poorer	26.7	32.3	14.2	76.9	26.3
Middle	28.8	40.3	26.1	71.9	34.3
Richer	38.2	58.6	36.1	75.6	45.7
Richest	41.2 ***	58.2 ***	45.1 ***	81.7 *	53.4 ***
**Comprehensive Knowledge of HIV**					
No	27.5	40.4	20.8	73.9	31.8
Yes	42.6 ***	59.4 ***	37.5 ***	84.7 ***	50.3 ***
**Number of Sexual Partners (excluding spouse) in the past 12 months**					
None	38.6	52.3	23.3	81.5	39.0
1 or more	26.5 ***	34.7 ***	28.0 **	67.9 ***	32.6 ***
**Sexually Transmitted Infection in Past 12 months**					
No	30.6	44.8	23.5	77.0	34.8
Yes	35.0	50.8	39.8 ***	79.1	49.3 ***

Note: Comprehensive knowledge assesses respondents’ understanding of HIV transmission and prevention.

* Represents p <0.05;

** represents p <0.01;

*** represents p <0.001

Results from adjusted logistic regression models are presented in Table 3. Associations between respondents’ characteristics and HIV testing were similar across all countries and within the pooled sample. Positive associations were evident between HIV testing and the following variables: age, age at sexual debut, and comprehensive knowledge of HIV. Youth aged 20–24 years had higher odds of HIV testing than youth aged 15–19 years (adjusted OR (aOR) = 2.19; 99% (1.99–2.40)). Compared to youth with age at sexual debut of less than 15 years, those who were older at sexual debut (15–18 years, or 19 years and above) had higher odds of HIV testing (aOR = 1.22; 1.07–1.39 and aOR = 1.47; 1.24–1.74 respectively). Also, those with comprehensive knowledge of HIV had higher odds of being tested for HIV compared to youth without comprehensive HIV knowledge (aOR = 1.98;1.76–2.22). Male youth had lower odds of being tested than female youth (aOR = 0.32; 0.28–0.37).

**Table 3:** Logistic Regression Models HIV testing by Participants' Demographic and Reproductive Health Characteristics.

	Congo^a^	Mozambique^a^	Nigeria^a^	Uganda^a^	Total^a^
	aOR^c^ (99% C.I.)	aOR^c^ (99% C.I.)	aOR^c^ (99% C.I.)	aOR^c^ (99% C.I.)	aOR^c^ (99% C.I.)
**Sex of Respondents**					
Female	*Ref*.	*Ref*.	*Ref*.	*Ref*.	*Ref*.
Male	**0.35 (0.25–0.50)**	**0.20 (0.16–0.26)**	**0.42 (0.34–0.51)**	**0.20 (0.14–0.28)**	**0.32 (0.28–0.37)**
**Age of Respondents**					
15 to 19 years	*Ref*.	*Ref*.	*Ref*.	*Ref*.	*Ref*.
20–24 years	**2.13 (1.70–2.67)**	**2.81 (2.34–3.38)**	**2.44 (2.06–2.89)**	**2.75 (2.07–3.66)**	**2.19 (1.99–2.40)**
**Age at Sex Debut**					
Less than 15 years	*Ref*.	*Ref*.	*Ref*.	*Ref*.	*Ref*.
15–18 years	1.16 (0.80–1.67)	0.96 (0.79–1.17)	**1.29 (1.03–1.60)**	**1.65 (1.12–2.42)**	**1.22 (1.07–1.39)**
19 years and above	**1.81 (1.00–3.26)**	1.36 (0.91–2.04)	**1.70 (1.32–2.20)**	**2.04 (1.20–3.47)**	**1.47 (1.24–1.74**
**Comprehensive Knowledge of HIV**^b^					
No	*Ref*.	*Ref*.	*Ref*.	*Ref*.	*Ref*.
Yes	**1.79 (1.29–2.48)**	**1.96 (1.61–2.39)**	**2.00 (1.66–2.42)**	**1.81 (1.28–2.56)**	**1.98 (1.76–2.22)**
**Number of Sexual Partners (excluding spouse) in the past 12 months**					
None	*Ref*.	*Ref*.	*Ref*.	*Ref*.	*Ref*.
1 or more	**0.45 (0.35–0.59)**	**0.23 (0.18–0.29)**	**0.46 (0.39–0.54)**	**0.38 (0.28–0.53)**	**0.39 (0.35–0.44)**
**Sexually Transmitted Infection in Past 12 months**					
No	*Ref*.	*Ref*.	*Ref*.	*Ref*.	*Ref*.
Yes	1.13 (0.80–1.60)	1.23 (0.91–1.67)	**1.70 (1.29–2.24)**	1.17 (0.83–1.65)	**1.56 (1.33–1.82)**

Notes:

^“a”^ Sample size: 4,482 Congo; 5,301 Mozambique; 10,492 Nigeria; 2642 Uganda; 23,367 Total

^“b”^ Comprehensive knowledge assessed respondents’ understanding of HIV transmission and prevention

^“c”^ Adjusted logistic regression models controlled for the effects of respondents' education, wealth index, and area of residence.

Statistically significant associations are depicted in bold font

### Cross country Comparison

As shown in Table 1, the prevalence of HIV testing varied across the countries; Uganda had the highest proportion of youth who had ever tested for HIV (77.5%) compared to Congo (31.4%), Mozambique (45.3%), and Nigeria (24.7%). Figures in Table 2 show that across all countries a consistent association was evident between uptake of HIV testing and each of the following variables: gender (p<0.0001), age (p<0.0001), and comprehensive knowledge of HIV (p<0.0001). Table 3 shows the adjusted logistic regression models for each country. History of an STI emerged as a country-specific predictor of HIV testing. Nigerian youth who reported an STI in the past 12 months had higher odds of HIV testing than those with no history of STI (aOR = 1.70; 1.29–2.24).

## Discussion

Many studies on HIV testing prevalence in sub-Saharan Africa have been based on samples of adults and pregnant women [10, 19, 20]. Thus, this study extends previous research that focused on populations of adults and/or pregnant women, and attempts to fill the gap in the literature by examining determinants of HIV testing among youths aged 15 to 24. The findings indicate that overall, only a quarter of youth demonstrated comprehensive knowledge of HIV/AIDS and just 4 in 10 had ever been tested for HIV. Sex and age of respondents were associated with HIV testing in all countries. Older respondents had higher odds of HIV testing than younger respondents (OR = 2.19) while males had lower odds of HIV testing than females (OR = 0.32).

The exception to low prevalence of HIV testing among youth was in Uganda where three-quarters of youth reported HIV testing. It is no surprise that Ugandan youth reported higher prevalence of HIV testing than other countries given the numerous efforts of the Ugandan government and international partners to prevent HIV transmission in the country following the devastating impact of HIV/AIDS on the country in the 1980s and early 1990s. Chief among HIV/AIDS prevention efforts in Uganda was the creation of the first AIDS Information Center on the African continent that provided anonymous and voluntary HIV counseling and testing (HCT) service to Ugandans [21]. Compared to other countries in this study, Uganda has a comprehensive and multi-sectorial policy and programmatic approach to curbing the spread of HIV. Uganda implemented detailed guidelines targeting most-at-risk populations for HIV and the general population. These guidelines allowed for: 1) Provider Initiated HIV Counseling and Testing (PITC); 2) Routine HIV testing and counseling in health centers; 3) Client-initiated voluntary counseling and testing; and 4) Community or home based HIV testing [22]. Similarly, Uganda is among few sub-Saharan African countries with a policy that provides a legal age of consent to HIV testing, which allows adolescents under the age of 18 years to assent to HIV testing without the additional consent of a parent or guardian. In Uganda, adolescents are able to consent to HIV testing and counseling services as early as 12 years of age [3]. The age of consent for HIV counseling testing is 16 in Congo and Mozambique. The legal age of consent in Nigeria is 18 although healthcare providers can use their discretion and not require parental consent if they believe the adolescent shows maturity and understanding of the process and potential results [3]. While many African countries have PITC services [23, 24], Uganda is one of the few that have successfully achieved high prevalence of HIV testing through community and home-based testing services [25, 26].

### Comprehensive knowledge of HIV and uptake of HIV testing among youth

This study revealed higher levels of HIV testing among youth with comprehensive HIV knowledge, an association that had been reported in other studies [27, 28]. To reduce the spread of HIV among youth, it is important to equip them with information about the virus. Unfortunately, the level of HIV knowledge among youth in sub-Saharan Africa is very low. This study found that between a quarter (Nigeria) to a third (Uganda) of youth demonstrated comprehensive knowledge of HIV. Other studies have reported similar low prevalence of HIV knowledge in Africa although there are variations by country [11, 27–29]. In rural Ethiopia, for example, less than half of young people (44% among boys and 41% among girls) correctly answered questions regarding HIV [29] while only 28.0% of Nigerian students aged 15–25 displayed comprehensive knowledge of the virus [28]. With less than 50% of sub-Saharan African youth demonstrating comprehensive knowledge of HIV, the region failed to meet the United Nations’ 2010 target of attaining factual HIV knowledge among 95% of young people aged 15–24 [28].

### History of STI and uptake of HIV testing

The present study revealed that about 1 in 10 youth had an STI, which if left untreated among this population in their reproductive age could lead to transmission to other sexual partners and newborns [30]. STIs, including herpes simplex virus type-2 and syphilis, cause ulceration and inflammation of genital area which lead to a breakdown of the genital mucosa, thus increasing susceptibility to HIV infection [31, 32]. Although studies across sub-Saharan Africa have shown the common occurrence of HIV and STI co-infection including syphilis, gonorrhea, chlamydia, and herpes-type2 [33–35], most of the evidence for HIV testing is targeted at women who attend antenatal care or people visiting tuberculosis clinics [10]. To reduce HIV infection therefore, the World Health Organization (WHO) recommends targeted HIV testing at STI testing and treatment centers [36].

### Age and gender differences

This study showed that older youth (20–24 years) had higher odds of HIV testing than younger youth (15–19 years). The present study also revealed that male youth had lower odds of HIV testing than females. The results of this study are consistent with the few available reports on gender and age differences in HIV testing among youth in sub-Saharan Africa. A study in Kenya and Zambia revealed that women aged 20 to 24 had higher odds of HIV testing than men of the same age group [37]. Further studies among Tanzanian students showed higher levels of HIV testing among those 18 years and older (48.5%) than students younger than 18 (24.4%). In this population also, male students had lower odds of HIV testing than female students [38]. Furthermore, women have a window of opportunity for HIV testing during antenatal care [39], so this targeted testing may explain their higher level of HIV testing. In HIV endemic countries, the WHO recommends universal HIV testing for all pregnant women and prompt treatment among HIV-positive women in order to prevent vertical transmission of the virus [36]. Thus, adoption operationalization of longstanding WHO guidelines coupled with national policies may add to the higher prevalence of HIV testing among females than males.

### Limitations

Our use of data generated from DHS surveys is a key limitation. This is because much of the data generated from the DHS is subject to recall bias, which may result in underreporting of HIV testing experiences, or inaccuracy of timing related to reported events. Additionally, the standard DHS used in this analysis does not provide test results of participants who reported taking an HIV test. Therefore, the study could not explore the relationship between the predictors of HIV testing and HIV test results. In addition, this study used data from different countries, collected over various years (2011–2013). At the time of this analysis, these surveys were the latest available data for countries included in the study. Nonetheless, comparing data across different years makes it challenging to conduct appropriate cross-country comparisons. It is equally important to note that this study did not present results adjusting for women's likelihood of receiving HIV test as part of their routine antenatal care. Nonetheless, the study explored the impact of testing for HIV during antenatal care on the other predictors of HIV testing. When women who may have tested for HIV as part of antenatal care were removed from the study sample, there was no significant difference in the observed associations in the pooled data. Furthermore, due to the design of DHS, women were over-represented in this study. The male questionnaire portion of the DHS items is limited to one male per household while all females of reproductive age within a household are included in the women’s questionnaire. Lastly, there are differences in national policies, cultures, and economic climates of the countries represented in this study. These differences may have contributed to the varying levels of HIV testing prevalence in the four countries investigated in this study.

## Conclusion

Successfully screening adolescents and young adults for HIV in most HIV-affected countries in sub-Saharan Africa is a public health priority. Local, national and international agencies should refocus efforts to promote routine HIV testing among youth. Additionally, as recommended by the World Health Organization, HIV testing should be integrated into routine STI screening and other health care services. Increased awareness and integrated healthcare efforts will help dispel myths about HIV/AIDS, promote HIV testing, and expedite the initiation of treatment options for youth who may already have contracted the virus. To foster better uptake of HIV testing among sub-Saharan African youth, it is important to employ a multipronged approach that removes barriers while providing opportunities for HIV testing in the community, at home, and at all levels of the health service. Finally, there is a need to re-examine contextual factors influencing HIV testing in sub-Saharan Africa, especially in situations where the age of consent for sexual activity for adolescents is lower than the age of consent for HIV testing and counseling. Research is needed to elucidate the effect of lowering the age of consent to HIV testing and counseling. Lesotho, South Africa, and Uganda have reduced the age of consent to HIV testing and counseling to 12 years and other countries (e.g., Botswana and Kenya) have removed age-limit requirement for consent to HIV testing and counseling [3]. Research is needed to understand the effect of these different approaches on uptake of HIV testing and counseling among adolescents, and on the protection of children’s rights.
